# Supplementary material for: The interactome of intact mitochondria by cross-linking mass spectrometry provides evidence for coexisting respiratory supercomplexes
Source: Mol Cell Proteomics. 2017 Dec 8;17(2):216–32. doi: 10.1074/mcp.RA117.000470 (PMC5795388; doi:10.1074/mcp.RA117.000470)
Supplement: Supplemental Data [file supp_RA117.000470_133922_0_supp_23978_fzffwf.zip › spectra_annotation/mito_DR_spectra_annotation/270-1-4-1-6-1.pdf]

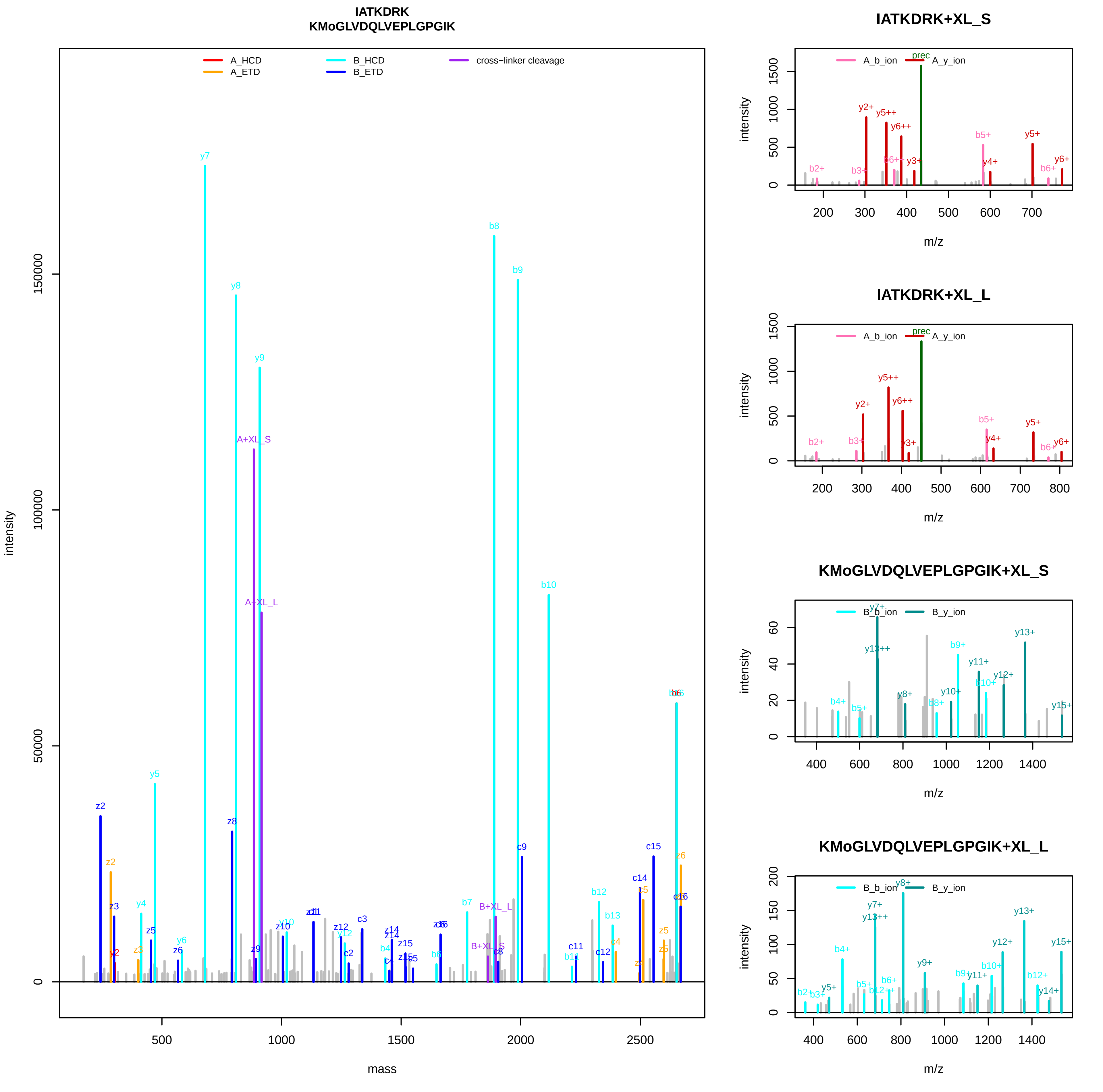
IATKDRK
KMoGLVDQLVEPLGPGIK
A_HCD
A_ETD
B_HCD
B_ETD
cross−linker cleavage
intensity
mass
0
50000
100000
150000
500
1000
1500
2000
2500
y7
b8
y8
b9
y9
A+XL_S
A+XL_L
b10
b6
y5
z2
z8
c9
c15
z6
z2
z3
y4
c14
b12
c5
b7
B+XL_L
c11
c3
z12
z14
z10
y10
b13
c4
z5
z9
c2
z15
B+XL_S
c8
b11
c11
c12
c16
IATKDRK+XL_S
A_b_ion
A_y_ion
prec
y2+
y5++
y6++
b5+
y5+
y3+
y4+
y6+
b6+
b2+
b3+
intensity
m/z
0
500
1000
1500
200
300
400
500
600
700
IATKDRK+XL_L
A_b_ion
A_y_ion
prec
y5++
y2+
y6++
b5+
y5+
y4+
b2+
b3+
y3+
b6+
y6+
intensity
m/z
0
500
1000
1500
200
300
400
500
600
700
800
KMoGLVDQLVEPLGPGIK+XL_S
B_b_ion
B_y_ion
y7+
y13++
y13+
b9+
y11+
y12+
b10+
y10+
y8+
b8+
b4+
b5+
y15+
intensity
m/z
0
20
40
60
400
600
800
1000
1200
1400
KMoGLVDQLVEPLGPGIK+XL_L
B_b_ion
B_y_ion
y8+
y7+
y13++
y13+
b4+
y12+
y15+
y9+
b10+
b9+
y11+
b12+
y5+
b5+
b6+
y14+
b2+
b3+
intensity
m/z
0
50
100
150
200
400
600
800
1000
1200
1400
